# Genetic diversity in aspen and its relation to arthropod abundance

**DOI:** 10.3389/fpls.2014.00806

**Published:** 2015-01-28

**Authors:** Chunxia Zhang, Barbara Vornam, Katharina Volmer, Kathleen Prinz, Frauke Kleemann, Lars Köhler, Andrea Polle, Reiner Finkeldey

**Affiliations:** ^1^College of Forestry, Northwest A&F UniversityShaanxi, China; ^2^Forest Genetics and Forest Tree Breeding, Büsgen-Institute, Georg-August-Universität GöttingenGöttingen, Germany; ^3^Forest Botany and Tree Physiology, Büsgen-Institute, Georg-August-Universität GöttingenGöttingen, Germany

**Keywords:** genetic variation, diversity experiment, *Populus*, SSRs, AFLPs, arthropod abundance

## Abstract

The ecological consequences of biodiversity have become a prominent public issue. Little is known on the effect of genetic diversity on ecosystem services. Here, a diversity experiment was established with European and North American aspen (*Populus tremula*, *P. tremuloides*) planted in plots representing either a single deme only or combinations of two, four and eight demes. The goals of this study were to explore the complex inter- and intraspecific genetic diversity of aspen and to then relate three measures for diversity (deme diversity, genetic diversity determined as Shannon index or as expected heterozygosity) to arthropod abundance. Microsatellite and AFLP markers were used to analyze the genetic variation patterns within and between the aspen demes and deme mixtures. Large differences were observed regarding the genetic diversity within demes. An analysis of molecular variance revealed that most of the total genetic diversity was found within demes, but the genetic differentiation among demes was also high. The complex patterns of genetic diversity and differentiation resulted in large differences of the genetic variation within plots. The average diversity increased from plots with only one deme to plots with two, four, and eight demes, respectively and separated plots with and without American aspen. To test whether intra- and interspecific diversity impacts on ecosystem services, arthropod abundance was determined. Increasing genetic diversity of aspen was related to increasing abundance of arthropods. However, the relationship was mainly driven by the presence of American aspen suggesting that species identity overrode the effect of intraspecific variation of European aspen.

## INTRODUCTION

In recent years, biodiversity is rapidly declining due to habitat loss and fragmentation driven by human activities including climate change (Smith et al., 2001). Thus, the ecological consequences of biodiversity losses have emerged as a prominent public issue (Loreau et al., 2001; Hooper et al., 2005). There has been much concern about the effect of species diversity on ecosystem functioning during the past decades. Numerous studies provide evidence that reduced species diversity has negative effects on ecosystem functions and services such as productivity, community stability, and trophic interactions (Tilman and Downing, 1994; Tilman et al., 2001; Cardinale et al., 2006; Scherber et al., 2010; Zavaleta et al., 2010; Haddad et al., 2011). However, less is known about the effects of genetic diversity within species on ecosystem functions although the role of genetic diversity as a key component of biodiversity is undisputed (Johnson and Stinchcombe, 2007).

Theory predicts associations between intraspecific (genetic) diversity and species diversity (Vellend, 2005) and an impact of genetic diversity on ecosystem functions (Johnson and Stinchcombe, 2007). Experimental evidence supporting strong relationships between intraspecific diversity and ecosystem processes is emerging (Bailey et al., 2009). Most experimental studies focused on grassland species with few clonal genotypes in each experimental replicate (Booth and Grime, 2003; Fridley and Grime, 2010). However, preliminary evidence suggests an impact of genetic diversity on the diversity of associated organisms and ecosystem processes also for long-living, sexually reproducing, woody perennials (Barbour et al., 2009). For example, genetic variation of a tropical tree species influenced the associated epiphytic plant and invertebrate communities in a complex tropical forest ecosystem and a positive relationship between the genetic distance of trees and community difference of the associated species were reported (Zytynska et al., 2011). Moreover, genetic diversity of cottonwood (black poplar) hybrids across eleven natural stands significantly correlates with arthropod diversity (Wimp et al., 2004). In common garden studies with European aspen (*Populus tremula* L.) from twelve populations, trait variation structured arthropod communities (Robinson et al., 2012). Furthermore, local poplar populations that were distinguished by microsatellite markers [simple sequence repeats (SSRs)] revealed correlations with the abundance of generalist leaf feeding insects (Kleemann et al., 2011). Overall, intertrophic interactions are important for ecosystem processes such as pollen and seed dispersal, nutrient cycling by herbivory, maintenance of habitat complexity, predation and food web interactions, thereby, regulating the provision of ecosystem services (Diaz et al., 2005). Knowledge on the influence of plant intraspecific diversity on their associated fauna that provide these services is scare.

The manipulation of intraspecific diversity is straightforward, if a limited and known number of different genotypes is clonally propagated and planted in pure or mixed experimental plots. However, most woody plant species reproduce sexually, therefore, studies based on the observation of different clonal diversity patterns in plant populations do not reflect the intraspecific diversity present in natural ecosystems dominated by woody species and do not allow to establish links between ecological functions and intraspecific diversity. Accordingly, there is an urgent need to better understand ecological effects of different intraspecific diversity patterns in experimental populations comprising sexually produced progenies (Hughes et al., 2008).

In the present study a diversity experiment was established comprising two closely related, inter-fertile aspen species (“trembling aspens”) of the section Leuce (Cervera et al., 2005): European aspen (*P. tremula*) and American aspen (*P. tremuloides* Michx.). To maximize the genetic diversity in this study progenies from seeds of single trees, population samples, and wildlings of aspen were planted. The plants originated from populations of different locations across Europe (Sweden, Poland, Germany, Austria, Switzerland) and the US. Because the genetic variation of a particular progeny array after sexual reproduction depends on numerous factors including the number of seed and pollen parents involved in the production of the planted progenies, we used the term “deme” in its original definition for an “assemblage of taxonomically closely related individuals” (Gilmour and Gregor, 1939) to distinguish different progeny arrays. In this sense, a deme is not necessarily equivalent to a specific taxonomic category such as a species, a subspecies or a variety (Gilmour and Heslop-Harrison, 1954), nor to a specific origin in the sense of, for example, a local interbreeding population (Winsor, 2000).

In the present study the demes from different locations were mixed in plots to obtain a design consisting of single demes and mixtures of two, four, and eight demes. Because of the unknown complexity of the intra-specific diversity in demes generated by open-pollination, an important goal of our study was the establishment of scales for the genetic diversity. We expected that our study design would result in increasing intra-specific diversity with an increasing number of demes in the mixture. Simple population genetic theory predicts that the diversity of a mixed plot will not be lower than the mean diversity of the demes contributing to this plot. However, the diversity of a specific combination of demes is difficult to predict, if demes differ with regard to their within-deme diversity and their differentiation from each other. This holds in particular if demes represent more than a single species as in our case. Thus, we tested whether the number of demes mixed in a plot is a proxy for its diversity. The analysis of a few hypervariable SSRs and a large number of dominant amplified fragment length polymorphisms (AFLPs) loci allows a comprehensive view on the neutral genetic diversity and differentiation (Vos et al., 1995; Mariette et al., 2001). In present study, based on the observation of genetic structures within each deme, our specific objectives were to investigate whether genetic diversities within the eight demes are significantly different from each other, and whether the genetic diversities of plots comprising a given number of demes (one, two, and four) are homogeneous, if only a single species (*P. tremula*) is included, and that the inclusion of a second species (*P. tremuloides*) increases the plot diversity.

*Populus* sp. are keystone species for a multitude of associated organisms (Whitham et al., 2006). In biomass plantations, usually clonal material is used and this genetic structure is vulnerable to infestation. Preceding studies have shown that trait variation affects the abundance of herbivores (Kleemann et al., 2011; Robinson et al., 2012). However, it is unknown whether intraspecific genetic variation as determined by neutral markers, is related to certain ecosystem functions such as the abundance of a functional ecological group such as the invertebrates. Therefore, we determined the abundance of different invertebrate groups in plots of different deme mixtures. To address the relationship between invertebrates and aspen intra-specific genetic diversity, the abundances of the animals were related to deme mixture (Shannon index by AFLP and expected heterozygosity by SSR). We assessed complex patterns of genetic diversity in a poplar experiment and related these results to invertebrate abundance in order to clarify the impact of mixing demes with different levels of diversity on creating genetic heterogeneity in a trial and to study the consequences of this heterogeneity on invertebrate abundance as an important ecosystem function.

## MATERIALS AND METHODS

### SITE DESCRIPTION

The poplar diversity experiment (POPDIV) was carried out at the Relliehausen Experimental Farm near Silberborn in the Solling Uplands, Germany (51°44′56′′N, 9°32′28′′E). The average annual temperature is 6.9°C while average annual rainfall is 1031 mm (Station Silberborn – Holzminden, Deutscher Wetterdienst 1961–1990). The site was situated on permanent, extensively grazed grassland at an altitude of 485 m above sea level. The soil of the experimental area is a shallow (40–60 cm), stony haplic Cambisol on middle Bunter (Triassic sandstone; Keuter et al., 2013).

### PLANT MATERIAL AND EXPERIMENTAL SET-UP

Eight aspen demes were used in this experiment: seven demes from Europe (*P. tremula*) and one from North America (*P. tremuloides*; for details, see **Table 1**). From May to June 2008 seeds were germinated and seedlings of most demes were cultivated in the greenhouse at 20°C and 16 h day length achieved by supplemental illumination. When reaching a height of about 5 cm, seedlings were planted individually in pots (13 × 13 cm) with nutrient-rich humus soil. Pots were placed outdoors and watered as necessary until autumn. With an average height of about 30 cm, the seedlings were out-planted in the experimental field at the end of October 2008. The aspen demes from Holstein, Germany and Bialystok, Poland, were provided by a tree nursery (Brunk Pflanzen, Elmshorn, Germany) as bare-rooted plants with a height of *ca*. 30 cm. The aspen trees from Sweden originated from natural regeneration on a clear-felled area close to Edsvalla and were collected within a privately owned forest area with permission given by the forest owner in October 2008.

**Table 1:** Description of the eight demes planted in the diversity experiment.

Country	Location	Abbreviation	Latitude	Longitude	Type of material	Type of collection	Source of plant material	Species
Germany	Göttingen	G2	51°31′N	9°58′E	Seeds	Single seed parent (known)	Institute of Forest Botany and Tree Physiology, University of Göttingen	*Populus tremula*
Germany	Göttingen	G8	51°31′N	9°58′E	Seeds	Single seed parent (known)		*P. tremula*
USA	Boston	USA	42°13′N	71°03′W	Seeds	Few seed parents (unknown)	Institute F. W. Schumacher Co.	*P. tremuloides*
Switzerland	Unterlunkhofen	CH	47°21′N	8°24′E	Seeds	Unknown	Swiss Federal Institute for Forest, Snow and Landscape Research	*P. tremula*
Germany	Holstein	G1	53°59′N	10°38′E	Seedlings	Population sample	Tree nursery (Brunk Pflanzen, Elmshorn, Germany)	*P. tremula*
Sweden	Edsvalla	S	59°26′N	13°12′E	Wildings	Population sample	Natural regeneration on a clear-felled area close to Edsvalla	*P. tremula*
Austria	Wienerwald	A	48°16′N	16°19′E	Seeds	Unknown	Woods close to Vienna	*P. tremula*
Poland	Białystok	PL	53°08′N	23°09′E	Seedlings	Population sample	Precise location unknown; tree nursery (Brunk Pflanzen, Elmshorn, Germany)	*P. tremula*

**FIGURE 1:**
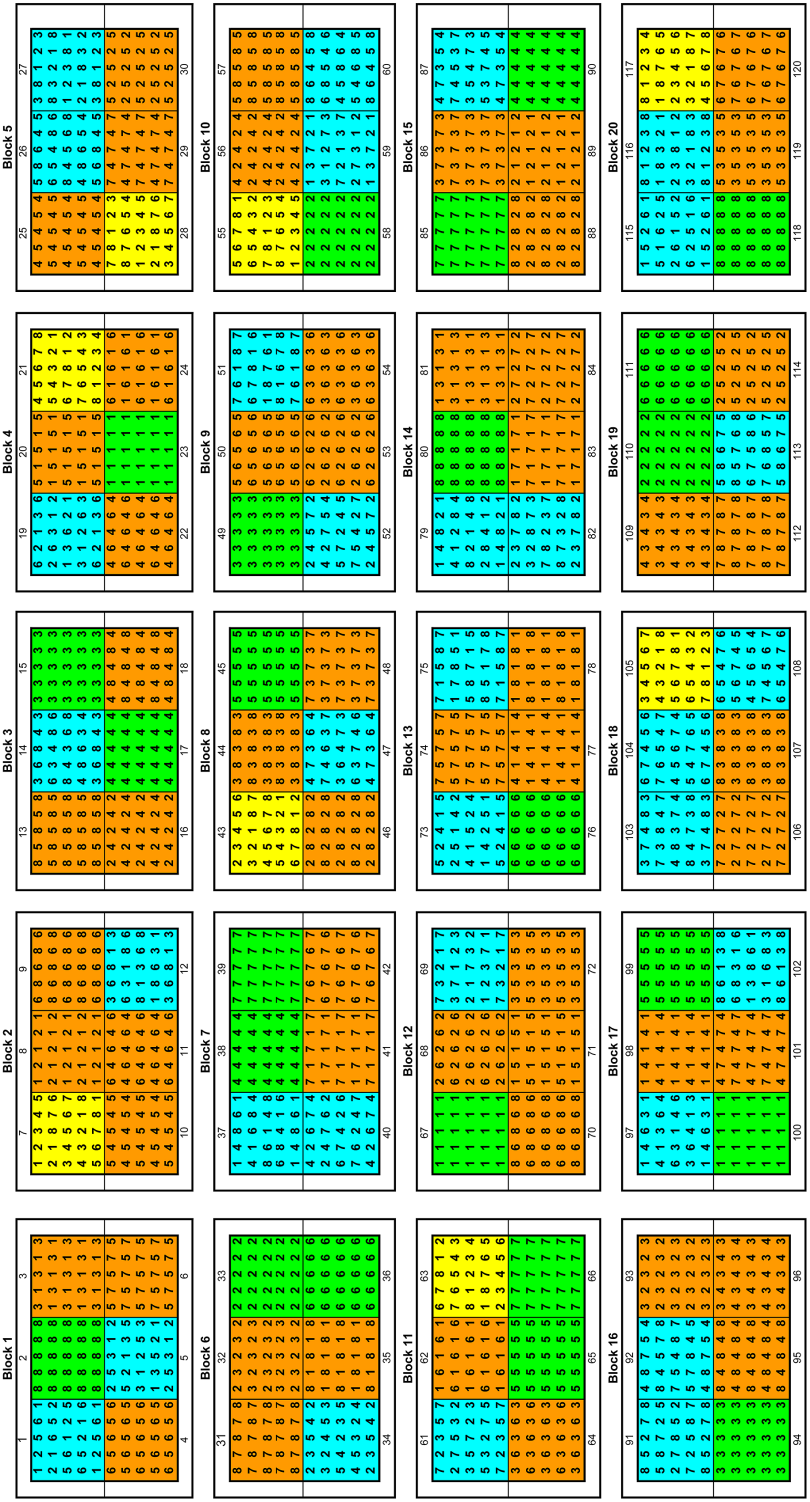
**Randomized block design of the poplar diversity.** The numbers (1–8) indicate the different demes included in the plot: 1-G2, 2-G8, 3-USA, 4-CH, 5-G1, 6-S, 7-A, 8-PL (the abbreviations are shown in **Table 1**). The plots in green, orange, blue, and yellow represent one, two, four, and eight demes in the plot, respectively.

Before the planting of the poplar seedlings the experimental site (110 × 170 m) was surrounded by a knotted-wire-netting-fence (game fence, height: 1.80 m). Inside the area, a second fence was installed with a height of 20 cm above-ground and reaching 70 cm below-ground to protect the plants from European Water Voles (*Arvicola amphibious*). Voles within the fenced area were decimated by traps (Topcat GmbH, L’Auberson VD, Switzerland) with permission of the lower nature conservation authority (administrative district Holzminden, Germany). Prior to the establishment of the poplar plants the biologically degradable herbicide Roundup (Monsanto, St. Louis, MO, USA) was applied, and the area was processed with a disk harrow. This is a typical treatment for plantation establishment and the herbicide dose was not toxic for arthropods (Safety data sheet, Monsanto Europe, version 5.0, 25.07.2011). The poplar seedlings were out-planted in a randomized block design with 20 blocks, each divided into six plots (**Figure 1**). In each plot, 25 poplar seedlings from up to eight different demes were planted at four levels: (1) each deme was planted solitary with three replications (24 plots), (2) two demes were planted in combination with each other (56 plots), (3) four demes were planted in random combinations (32 plots) and (4) all demes were planted in combination (eight plots). The total number of experimental trees was 3000, and the distance between the trees was 1.5 m. To avoid edge effects each block was surrounded by a row of additional trees (1080 plants in total) that was not taken into account for the measurements and analyses.

### PLANT MATERIAL AND DNA EXTRACTION

Fresh leaves were collected from all seedlings planted in the plots comprising single demes and eight demes in the POPDIV experimental field in spring 2009 (**Figure 1**). If some seedlings were missing in these plots, additional samples were collected from plots comprising two demes (**Figure 1**). Total genomic DNA was extracted with the DNeasy Plant Minikit (Qiagen, Hilden, Germany) according to the manufacturer’s protocol.

### MICROSATELLITE ANALYSES

The microsatellite markers *WPMS14*, *WPMS16* (Smulders et al., 2001), *PTR*2, *PTR4* (Dayanandan et al., 1998), *PTR5*, *PTR6*, *PTR8,* and *PTR14* (Rahman and Rajora, 2002; Suvanto and Latva-Karjanmaa, 2005) were used for PCR amplification (**Table 2**). The forward primer of each marker was labelled with 5-HEX (5′-hexachlorofluorescein) or 6-FAM (6′-carboxyfluorescein). The linkage groups of markers used in this study are known according to genetic linkage map in aspen with the exception of *PTR6* (Pakull et al., 2009). The gene loci located on different chromosomes were assumed to be independently inherited. Three multiplex PCR reactions were developed and routinely used in our analyses (**Table 2**).

**Table 2:** Description of the nuclear microsatellite markers used in this study.

Multiplex	Microsatellite markers	Label	Annealing temperature (°C)	Linkage group	Observed range (bp)	A
1	PTR2	Fam	54	9	190–240	12
	PTR5	Fam		11	130–150	11
2	PTR6	Hex	55	Scaffold_127	95–130	18
	PTR8	Hex		8	120–150	19
	PTR14	Hex		3	150–220	15
3	PTR4	Hex	57	3	100–130	10
	WPMS14	Fam		5	200–287	19
	WPMS16	Fam		7	150–200	13

*“A” refers to the number of alleles in the total sample of this study. Linkage groups for PTR2, PTR5, PTR8, PTR14, PTR4, WPMS14 and WPMS16 were according to Pakull et al. (2009).*

The PCR reaction was performed in a total volume of 15 μl containing 1 μl of genomic DNA (∼10 ng), 1.5 μl of 10 × reaction buffer (0.8 M Tris-HCl pH 8.7, 0.2 M (NH_4_)_2_SO_4_, 0.2% w/v Tween-20; Solis Biodyne), 1.0 μl of MgCl_2_ (25 mM), 1.0 μl of each dNTP (2.5 mM, Fermentas), 1 μl S-solution (Solis Biodyne), 1 μl of each primer (5 pmol/μl) and 0.2 μl of *Taq* DNA polymerase (5 U/μl, Solis Biodyne). The PCR reaction was carried out in a Peltier Thermal Cycler (PTC-200, MJ research) with the following steps: 95°C initial denaturation for 15 min followed by 35 cycles at 94°C for 1 min (denaturation), annealing for 1 min (the annealing temperature was changed according to different markers; **Table 2**) and at 72°C for 1 min (extension). In the last cycle the extension period was increased to 10 min at 72°C. PCR products were diluted in appropriate proportions according to the band intensity on agarose gels and denatured at 95°C for 2 min in HiDi^TM^ formamide containing a 500-bp ROX standard (Applied Biosystems, Foster city, CA, USA). Fragments were separated on ABI Prism 3100 genetic analyser (Applied Biosystems, Foster city, CA, USA). The size of the fragments was determined with the software packages GeneScan 3.7 and Genotyper 3.7 (Applied Biosystems, Foster city, CA, USA).

### AFLP ANALYSES

The AFLP technique followed the protocol of Vos et al. (1995) with minor modifications. The total genomic DNA was digested with *Eco*RI and *Mse*I. Double-stranded *Eco*RI and *Mse*I adaptors were ligated to the ends of the restriction fragments to generate the template DNA for pre-selective amplification. The restriction and ligation were performed overnight at room temperature. The pre-selective amplification was carried out with diluted DNA from the ligation reaction and the primer pairs *Eco*RI-A and *Mse*I-G. The reactions were programmed to start at 72°C for 2 min, 20 cycles each at 94°C for 10 s, at 56°C for 30 s, at 72°C for 2 min and finally at 60°C for 30 min. The selective amplifications were carried out using diluted pre-selective amplification products and the primer pairs *Eco*RI-ACA with both *Mse*I-GAAA and *Mse*I-GAAT. *Eco*RI-ACA was labeled with the fluorescent dye 6-FAM. The reaction was programmed to start at 94°C for 2 min, 10 cycles each consisting of denaturation at 94°C for 10 s, annealing at 65°C for 30 s (subsequently reduced by 1°C per cycle) and extension at 72°C for 2 min, and continued with 24 cycles with the following temperature profile: 94°C for 10 s, 56°C for 30 s and 72°C for 2 min, ending with a final extension step at 60°C for 30 min. The fragments were analyzed similarly to the SSR method described in detail above.

### GENETIC DIVERSITY AND DIFFERENTIATION OF THE EIGHT DEMES

The multilocus genotypes based on SSR and AFLP markers were compared for each deme by using the multilocus matches function in GenAlEx version 6.41 (Peakall and Smouse, 2006) in order to identify clones, and all the multilocus genotypes were carefully checked visually and only identical mutlilocus genotypes were considered as clones in this study. For SSR markers, the numbers of alleles per locus (A) and private alleles (A_P_), observed and expected heterozygosity (H_o_, H_e_) as well as the fixation index (F) were calculated by the program genetic data analysis (GDA) version 1.1 (Lewis and Zaykin, 2001). We defined an allele as private if it occurred only in one specific deme and did not appear in the other demes. The pairwise differentiation between demes was assessed by the θ-statistics of Weir and Cockerham (1984), and the significance was tested using the log-likelihood statistics G implemented in FSTAT version 2.9.3.2 (Goudet, 2001). Significance values were calculated after sequential Bonferroni correction with 6000 permutations (Rice, 1989). An analysis of molecular variance (AMOVA) was performed using the program ARLEQUIN version 3.5.1.2 (Excoffier and Lischer, 2010), and the variance distribution was estimated according to Weir and Cockerham (1984). Nei’s (1972) genetic distances were calculated using the program Populations version 1.2.30 (Langella, 1999) with 1000 permutations and used to construct dendrograms by the unweighted pair group method with arithmetic mean (UPGMA) algorithm viewed in TreeView (Page, 1996).

For AFLP markers, reproducibility was carefully and repeatedly tested by the selection of primers showing high degrees of reproducibility in preliminary tests with multiple samples from all demes and by including a single genotype as positive control in all routinely scored plated consisting of 96 samples. The amplified fragments within a range of 75–500 bp were scored manually as “1” and “0” for its presence and absence, respectively. If a lack of reproducibility was observed at any fragment, this fragment was excluded from further analyses. Only reproducible and polymorphic loci with clearly amplified fragments were used for data analysis. Moreover, the whole data set was checked for private alleles (unique to one given deme, A_P_). The number of polymorphic loci (PL), percentage of polymorphic loci (PPL) and Shannon index (I) were calculated using the program POPGEN version 1.32 (Yeh et al., 1997). An AMOVA was performed based on the pairwise squared Euclidean distances among molecular phenotypes of bands using the program ARLEQUIN version 3.5.1.2 (Excoffier and Lischer, 2010) to partition the genetic diversity (Excoffier et al., 1992). The pairwise F_ST_ values according to Weir and Cockerham (1984) obtained from AMOVA were used to assess the genetic differentiation between demes and the significance of the pairwise F_ST_ values was tested based on the calculation of probability values after 10,100 permutations.

For AFLP markers, the genetic similarity between demes was defined as the averaged simple matching coefficients (Sokal and Michener, 1958) between individuals within demes calculated using the package scrime (Schwender, 2007) in R^1^. The genetic distances between demes defined as one minus genetic similarity were bootstrapped with 1000 permutations conducted in R^2^. The genetic distances were analyzed based on the majority rule using the Consensus program in PHYLIP version 3.69 (Felsenstein, 2005) to construct the dendrogram using the UPGMA algorithm in TreeView (Page, 1996).

### GENETIC DIVERSITY OF EACH PLOT IN THE EXPERIMENT

Genetic diversities of each plot were calculated based on the assumption that the seedlings of a particular deme planted in a given plot are a random sample of the seedlings of this deme. This assumption is reasonable since seedlings were randomly planted in pots in the greenhouse and later in the field. The expected allele frequencies of each plot were calculated for each locus separately as weighted means of the allele frequencies of the demes included in a plot with the weights equivalent to the relative proportion of a deme in a plot. Deviations of actual from calculated allele frequencies of plots are possible at single loci due to sampling effects, but the impacts on overall multilocus diversities are negligible.

For SSR markers, the genetic diversity of the plots was estimated as expected heterozygosity. For AFLP markers, the genetic diversity of the plots was assessed as Shannon index based on the frequency of band presence and absence as implemented in AFLPSURV version 1.0 (Vekemans, 2002). The band frequency of each marker for the samples included in the plot was calculated according to their weighted means. For each locus, the genetic diversity was estimated as: -(*p_i_lnp_i_*+*p_j_lnp_j_)*, where *p_i_* and *p_j_* is the frequency of band presence and absence, respectively (Lewontin, 1972). The genetic diversity of the plots was averaged across all loci.

An ANOVA was conducted to test if differences between the average diversity (expected heterozygosity for SSRs; Shannon index for AFLPs) of plots with different numbers of demes were statistically significant. To test the null hypothesis for the comparison of measured and predicted diversity, the sign rank test was applied (Statgraph Centurion; Mo, USA).

### INVERTEBRATE COLLECTION

Invertebrates were collected on sunny days in the last 2 weeks in May 2012. For this purpose a standardized technique was applied by hitting each stem five times with a stick and collecting the detached animals in a white umbrella-shaped tray at the bottom (Jervis, 2005). The animals were shaken down from the nine central trees in each of the 120 plots. The invertebrates of each plots were pooled resulting in *n* = 120 samples. The animals were stored in 70% ethanol. The number of collected invertebrates per plot was counted and sorted according to the categories: caterpillars, beetles, flies, and spiders. Tree height was measured. For statistical analyses rank correlations (Spearman’s D, Spearman’s r_s_, and P with the Monte Carlo permutation test with 9999 random replicates) were calculated for the relationship between H_e_ by SSR markers and the abundances of the invertebrates using the free PAST software package 2.17c^2^ (Hammer et al., 2001). To test for differences between deme mixtures the Kruskal Wallis test was employed using the PAST software 2.17c.

## RESULTS

### PLOIDY AND CLONAL STRUCTURES

We observed a maximum of two alleles at all investigated SSR loci in single individuals. Thus, we assume that all investigated plants were diploid. Nine clones were jointly identified by both SSR and AFLP markers within one *P. tremula* deme which had been collected from wildlings grown on a clear felled area in Sweden (**Table 1**). Most of the clones were observed in two plots comprising only this Swedish deme (**Figure 1**, plots 36 and 111), suggesting an uneven distribution of the clones within the experimental plots. However, among a total of 99 plants only 23 plants were discovered that belonged to 9 clones that the contribution of clones was below 15% for this deme.

### GENETIC DIVERSITY WITHIN ALL DEMES

Moderate to high levels of genetic diversity were observed within all demes at SSR loci (**Table 3**). All SSR loci used in this study were moderately or highly polymorphic within all demes with at least 10 different alleles observed in the complete data set (**Table 2**). The average number of alleles per locus varied from 5.3 to 8.8 within the German (G8) and Swedish demes, respectively. The observed and expected heterozygosities indicated the highest diversity within the North American *P. tremuloides* deme. Most private alleles were also observed in this deme. The lowest diversity was estimated for the *P. tremula* deme G8, which was a progeny array from a single seed tree growing in Göttingen, Germany. Fixation indices (F) were calculated for all demes, although Hardy-Weinberg structures are not biologically meaningful genotypic reference structure for the single tree progenies. Mean *F* values were low and moderately positive for the population samples (0.083) and in the case of the single mother tree progeny G8 even slightly negative.

**Table 3:** Genetic diversity of eight aspen demes.

Deme	SSRs						AFLPs				
	N	A	A_P_	H_e_	H_o_	F	N	A_P_	PL	PLP	I
G2	100	8	3	0.580	0.548	0.056	100	0	86	0.528	0.192
G8	100	5.3	1	0.398	0.401	-0.008	98	0	67	0.411	0.122
USA	97	8.6	20	0.710	0.647	0.090	99	38	107	0.656	0.238
CH	100	7.8	3	0.475	0.456	0.039	97	1	84	0.515	0.185
G1	100	6.4	1	0.484	0.452	0.066	99	1	77	0.472	0.192
S+	99	8.8	3	0.652	0.543	0.169	98	1	87	0.534	0.254
S-	76	8.8	3	0.665	0.558	0.163	75	1	87	0.534	0.219
A	99	6.5	3	0.482	0.412	0.145	95	2	75	0.460	0.170
PL	100	7.6	1	0.616	0.549	0.110	99	3	85	0.522	0.199
Total	773	14.6	35	0.637	0.501	–	762	46	163	1	0.299

*N, sample size; A, mean number of alleles per locus; A_P_, number of private alleles; H_o_, observed heterozygosity; H_e_, expected heterozygosity; F, fixation index; PL, number of polymorphic loci; PLP, proportion of polymorphic loci; I, Shannon index. For the Swedish deme (S), diversity parameters were calculated including (S+) or excluding clonal replicates (S–).*

**FIGURE 2:**
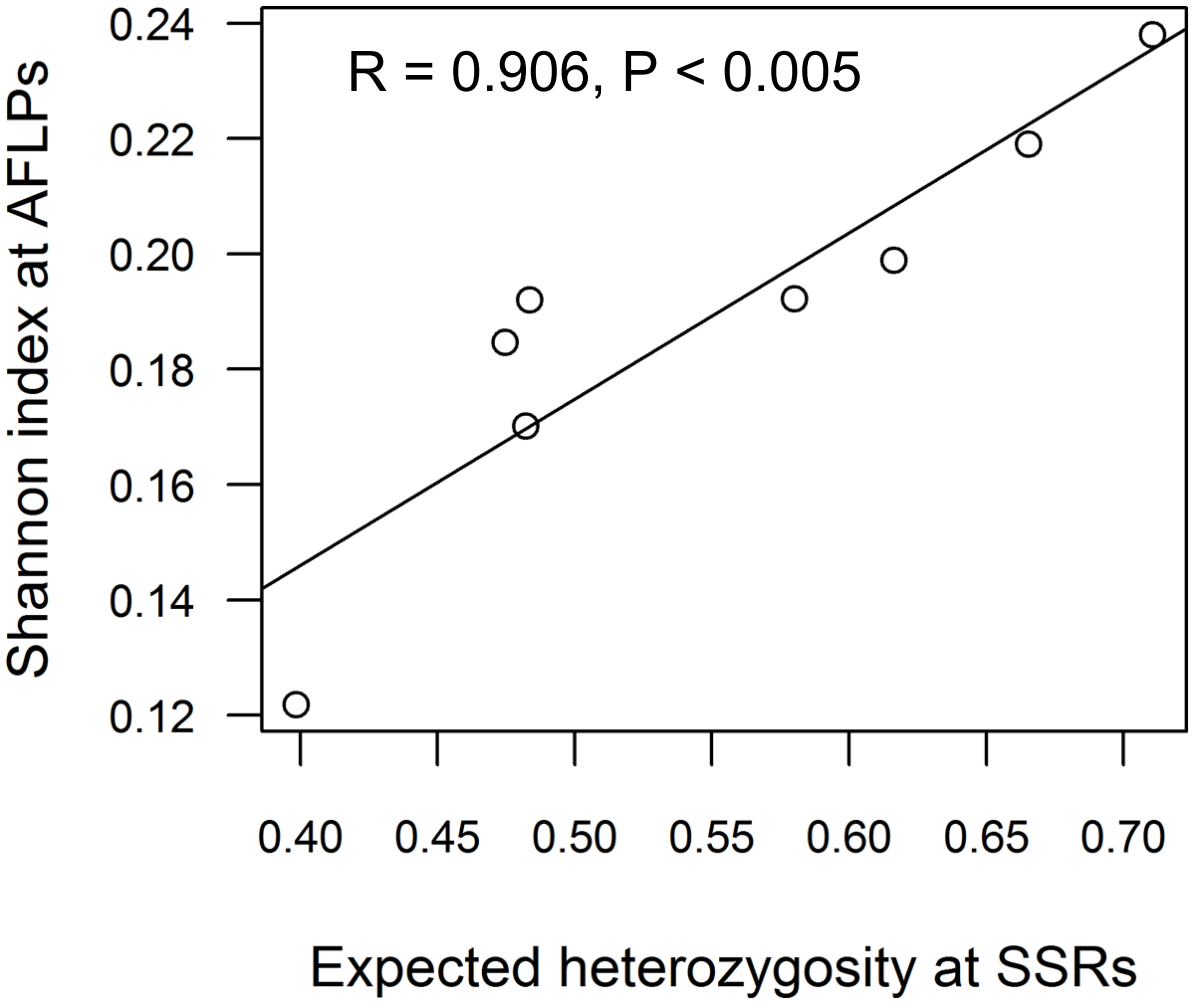
**Correlation of genetic diversity estimates from SSRs (expected heterozygosity) and AFLPs (Shannon index) across eight aspen demes.** For the Swedish deme, diversity estimates were used for Pearson correlation analysis after removing the clonal replicates (see **Table 3** S–, for details).

The two AFLP primer pair combinations yielded 163 clear and repeatable peaks in the range of 75–492 bp. By far most private alleles were present within the North American *P. tremuloides* deme. The observation of the maximum genetic diversity within the American *P. tremuloides* deme and the minimum genetic diversity within deme G8 was confirmed at the AFLP markers based on all analyzed parameters including the number of polymorphic loci and Shannon indices (**Table 3**). The Pearson correlation between the expected heterozygosity at SSR markers and Shannon indices at AFLPs was highly significant (*R* = 0.906, *P* < 0.005, **Figure 2**).

### GENETIC DIFFERENTIATION AMONG DEMES AND GENETIC DISTANCE BETWEEN DEMES

The *F*_ST_ values of all pairs of demes showed significant differentiation of the eight demes analyzed here by SSR markers (**Table 4**, above diagonal) and AFLP markers (**Table 4**, below diagonal). For SSR markers, the *F*_ST_ values varied from 0.031 to 0.308 with an average of 0.150, indicating that about 15% of the genetic variation was present among demes. Strong genetic differentiations were observed between the North American *P. tremuloides* deme and the European *P. tremula* demes (0.16 < *F*_ST_ < 0.31). Most *P. tremula* demes were also moderately to highly differentiated. High genetic differentiation was confirmed between the North American *P. tremuloides* deme and the European *P. tremula* demes by AFLP markers, which indicates strong genetic differentiation between different species (0.55 < *F*_ST_ < 0.63). Moderate genetic differentiation was also found among the *P. tremula* demes (0.05 < *F*_ST_ < 0.15).

**Table 4:** Pairwise *F*_ST_ values between the eight demes by SSR (above diagonal) and AFLP markers (below diagonal).

	G2	G8	USA	CH	G1	S	A	PL
G2		0.182*	0.233*	0.169*	0.102*	0.059*	0.121*	0.080*
G8	0.316*		0.308*	0.304*	0.134*	0.143*	0.169*	0.156*
USA	0.574*	0.642*		0.220*	0.270*	0.172*	0.249*	0.167*
CH	0.220*	0.284*	0.560*		0.151*	0.107*	0.152*	0.131*
G1	0.181*	0.244*	0.588*	0.159*		0.057*	0.073*	0.088*
S	0.122*	0.213*	0.566*	0.129*	0.083*		0.069*	0.031*
A	0.243*	0.345*	0.602*	0.239*	0.212*	0.148*		0.091*
PL	0.176*	0.209*	0.582*	0.129*	0.125*	0.078*	0.168*	

*significant at *P* < 0.001.

For both markers, the AMOVA analysis at two hierarchical levels showed that most of genetic variation was present within demes (84% at SSRs and 63% at AFLPs, **Table 5**). The AMOVA revealed high and significant differentiation among demes at both SSRs and AFLPs (*P* < 0.001). The AFLP markers showed a higher genetic differentiation (36.8%) compared to SSR markers (16.0%).

**Table 5:** Analysis of molecular variance (AMOVA) test to partition the total diversity at AFLPs and SSRs.

	Source of variation	Df	SQ	VC	POV	*F*_ST_	*P* value
SSR	Among demes	7	575.790	0.413	16.010	0.160	<0.001
	Within demes	1544	3347.013	2.168	83.990		
	Total	1551	3922.802	2.581			
AFLP	Among demes	7	3893.575	5.741	36.770	0.368	<0.001
	Within demes	754	7442.811	9.871	63.230		
	Total	761	11336.386	15.613			

*df, degree of freedom; SQ, sum of sequares; VC, variance components; POV, percentage of variation; *F*_ST_, Fixation indices, *P* values were obtained after 10,000 permutations.*

**FIGURE 3:**
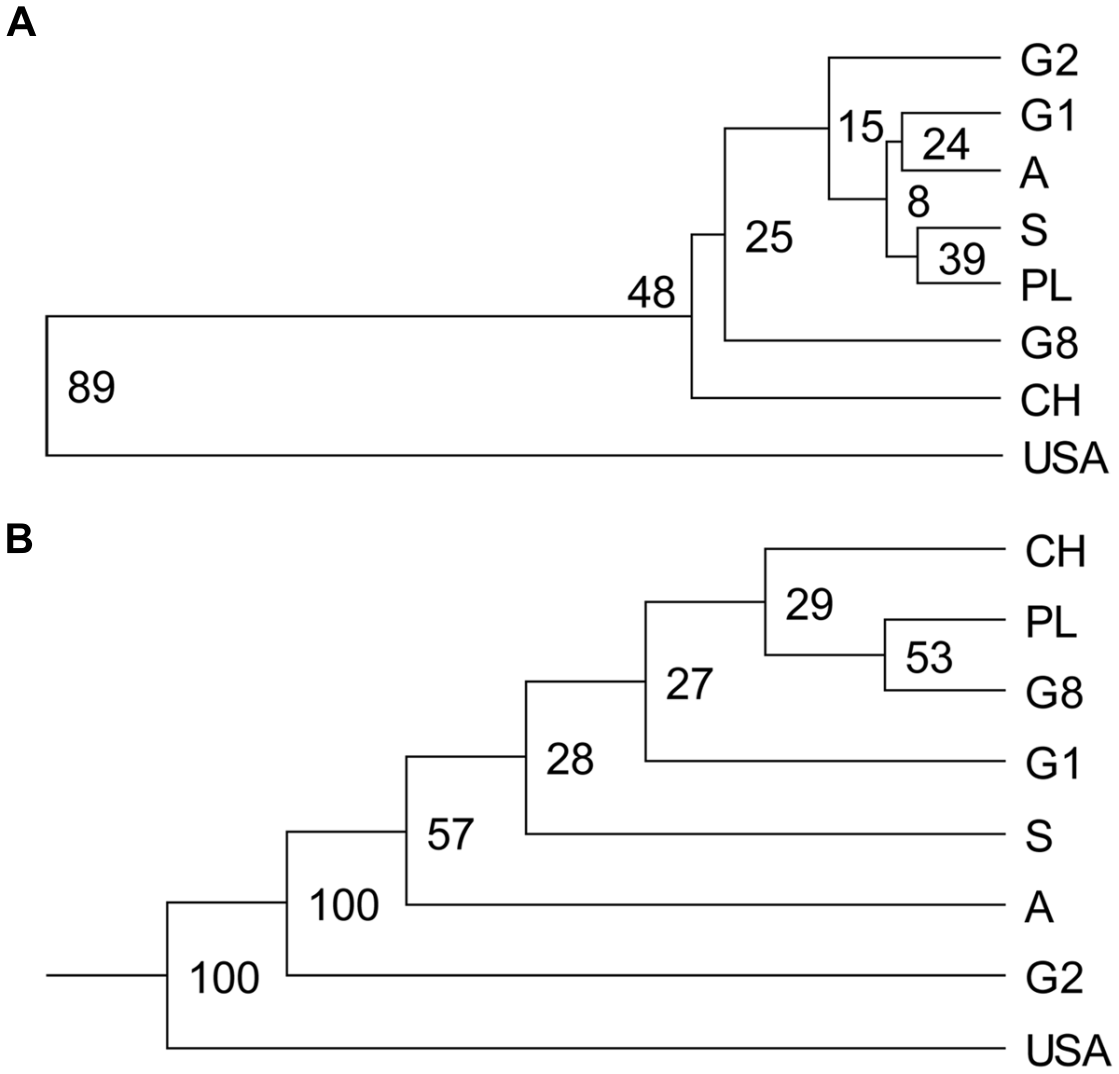
**Unweighted pair group method with arithmetic mean dendrograms based on genetic distances calculated from both SSRs and AFLPs. (A)** Nei’s (1972) genetic distances based on SSRs between demes was used to construct the UPGMA dendrogram. **(B)** Consensus UPGMA dendrogram was drawn based on genetic dissimilarity between demes (genetic similarity based on simple matching coefficient) calculated from AFLPs. Numbers at nodes are percentage over 1000 bootstrap replicates. Abbreviations for the demes are as in **Table 1**.

For both SSR and AFLP markers, the genetic distances between the eight demes were visualized in UPGMA dendrograms (**Figure 3**). Congruent results were obtained from both markers. Large genetic distances were observed between the North American *P. tremuloides* deme and the European *P. tremula* demes resulting in clear outgrouping of the North American *P. tremuloides* deme from the European *P. tremula* demes with high bootstrap support (89% at SSRs and 100% at AFLPs). This finding confirmed that *P. tremuloides* represents a genetically distinct species in comparison to the European *P. tremula* demes.

### WITHIN PLOT GENETIC DIVERSITY

Because not all plots could be measured, the overlap of the estimation of the genetic diversity with measured values was analyzed. We predicted the diversity in 8-deme plots with measured diversity of the individual demes and compared these values with the measure diversity in the 8-deme plots. We found a strong match between the predicted and measured values (**Table 6**). While the overlap was almost 100% for He, the AFLP markers slightly, but consistently overestimated the diversity (6.7%) of the 8-deme plots (**Table 6**).

**Table 6:** Validation of measured and predicted genetic diversity.

	He by SSR	Shannon index by AFLP
Index (measured)	0.641 ± 0.007	0.278 ± 0.005
Index (predicted)	0.639 ± 0.002	0.297 ± 0.002
Difference (%)	0.3	6.7
Large sample test statistic	0.911	2.313
*P*	0.362	0.021

**FIGURE 4:**
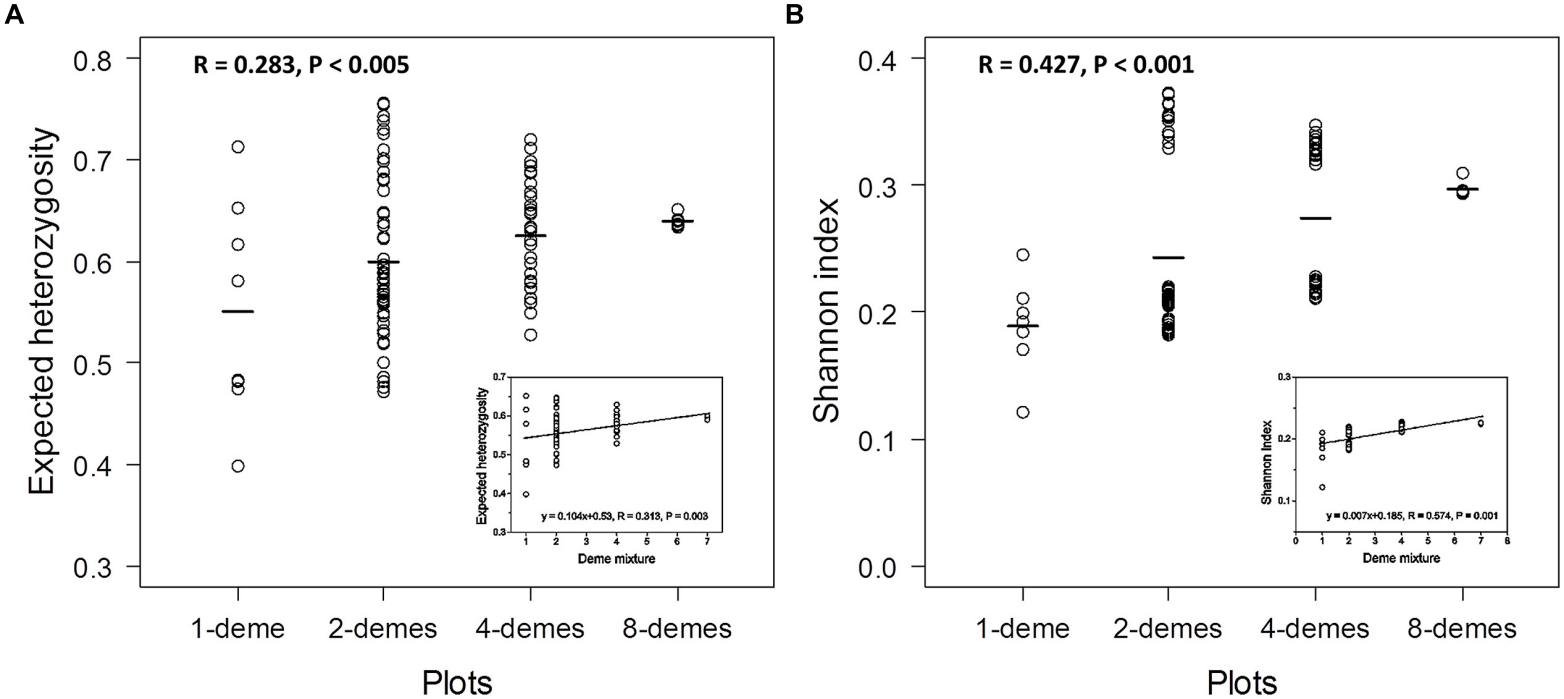
**Genetic diversity of all plots in the polar diversity experiment at SSRs and AFLPs.** The calculation for SSR loci was based on the expected allele frequencies with weighted mean method **(A)** and AFLP markers based on the band presence and absence with weighted mean method **(B)**. The open circles represent the genetic diversity of plots according to the four levels: 1-deme, 2-demes, 4-demes, and 8-demes. The lines represent the average diversity value of each level. Insets: Intraspecific genetic diversity of *P. tremula* with increasing deme mixture.

The genetic diversity was calculated for all plots in our study based on the analyses of both markers. The diversity measures were grouped according to the number of demes present within the plot: 1-, 2-, 4-, and 8-demes (**Figure 4**). The genetic diversity within plots composed of 1, 2, or 4 demes was highly variable (**Figure 4**) because the genetic diversity within a given deme and among the demes was not homogenous (**Table 3**). Because the plots with eight demes contained the plants of all demes in an almost balanced manner (three plants of each of the eight deme + 1 extra plant of any deme = 25 plants), the 8-deme plots showed only very little variation (**Figures 4A,B**). The mean genetic diversity increased with increasing number of different demes included within plots as expected. The mean diversities of plots with different number of demes were significantly different from each other based on the ANOVA test both for AFLPs and SSRs (**Figures 4A,B**). However, it was notable that the absolute maximum of genetic diversity was present in 2-deme plots and that the Shannon indices on 2- and 4-deme plots clearly separated plots with high (>0.30) and plots with low (<0.22) indices (**Figure 4B**). The reason for this separation was the presence of *P. tremuloides* in these combinations, which resulted in much higher genetic diversity than in plots containing only different *P. tremula* demes (**Figure 4B**). Because *P. tremuloides* strongly affected the analysis, we determined the relationship between neutral genetic diversity measures and number of demes in the mixtures for plots comprising only *P. tremula* demes and for the 8-deme plots without the USA deme. Increasing deme diversity resulted in increasing genetic diversity (**Figure 4**, inset). However, the increment was small, regardless of whether expected heterozygosity or Shannon indices were used as the genetic measure.

### PLOT DIVERSITY AND INVERTEBRATE ABUNDANCE

The invertebrate inventory in the aspen plots showed that beetles formed the largest group (mean number per plot ± SE: 11.3 ± 0.9), whereas all other groups occurred with much lower abundances (spiders: 1.9 ± 0.2, caterpillars: 1.7 ± 0.1, flies: 1.6 ± 0.2). The mean total number of invertebrates on the central trees per plot was 17.0 ± 1.0 and their density amounted to 13.0 ± 0.6 animals per meter of plant height.

**FIGURE 5:**
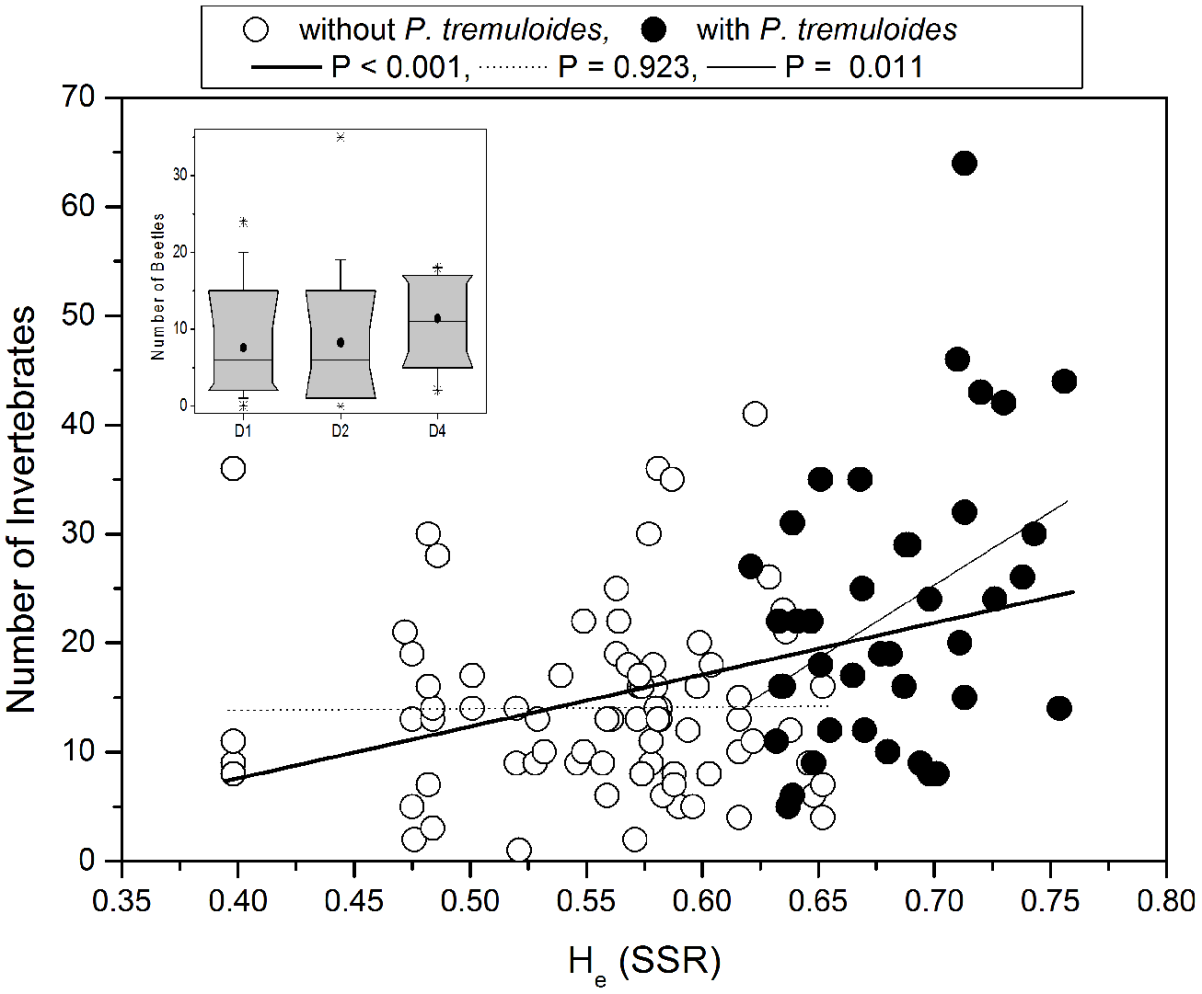
**Invertebrate abundance in relation to the expected heterozygosity (He) measured by SSR markers.** Data were fitted by linear regression. The abundance of arthropods was determined for each plot. Open symbols: plots containing only *P. tremula* demes, closed symbols: plots containing *P. tremuloides* demes. Inset: Beetles abundance in 1-, 2-, and 4-deme plots with *P. tremula*. Data are shown as box plots indicating the 10-, 25, 50, and 90% range of the data. In the boxes the vertical line indicates the median and black circle the mean.

To investigate potential relationships between the genetic diversity and the abundance of invertebrates we correlated expected heterozygosity (He) with invertebrate abundance. Since our previous analysis showed a strong effect of *P. tremuloides* on the genetic diversity indices, rank correlation analyses were conducted for the detected invertebrate groups for all plots and additionally only for the plots without the USA deme (**Table 7**). When all plots were included we found that He was significantly correlated with beetle abundance (positive) and with caterpillar abundance (negative; **Table 7**). The relationship between He and total invertebrate abundance was also significant and positive (**Figure 5**). However, when plots with and without *P. tremuloides* were analyzed separately, it was apparent that invertebrate abundance was unrelated to *P. tremula* diversity and that the observed increase was driven by the presence of *P. tremuloides* (**Figure 5**). When the correlation analysis was conducted with the Shannon indices of the AFLP markers for all plots, the relationship with invertebrate abundance was also highly significant (*R* = 0.35, *P* < 0.001) and disappeared when *P. tremuloides* was removed from the analysis. Correlations were neither detected when the invertebrate groups (spiders, beetles, caterpillars, and flies) in plots with and without *P. tremuloides* were analyzed separately (**Table 7**).

**Table 7:** Rank correlation analysis for invertebrate abundance with the expected heterozygosity (He) of the SSR markers.

	D	*p* (perm)	Rs	*p* (perm)
**All demes**
Spiders	252620	0.3533	0.0856	0.3533
Caterpillars	342990	**0.0151**	-0.2224	**0.0151**
Beetles	176020	**0.0001**	0.3877	**0.0001**
Flies	274390	0.9794	0.0023	0.9794
Invertebrates (all)	196780	**0.0006**	0.3159	**0.0006**
**Only *P. tremula***
Spiders	79956	0.8175	-0.0264	0.8175
Caterpillars	95149	0.1043	-0.1864	0.1043
Beetles	73695	0.3768	0.0995	0.3768
Flies	80230	0.8431	-0.0225	0.8431
Invertebrates (all)	82515	0.9538	-0.0066	0.9538

*perm, permutation. Bold numbers indicate significant p values.*

We furthermore tested the effect of deme diversity on invertebrate abundance. No significant differences were observed for any of the invertebrate groups in different demes when the deme USA was present. When only plots differing in the genetic diversity of *P. tremula* were included in the analysis, we found that beetles were slightly more abundant in 4-deme plots than in 1- or 2-deme plots (after Bonferoni correction: *p* ≤ 0.05; **Figure 5**, inset).

## DISCUSSION

### GENETIC DIVERSITY OF THE EIGHT DEMES

Both SSR and AFLP markers are commonly used to examine genetic diversity (Cao et al., 2006; de Jesus et al., 2013; Emanuelli et al., 2013). Usually, SSR markers show higher levels of genetic diversity than those revealed by AFLP markers due to marker specific characters: SSR markers are codominant and highly polymorphic while AFLP markers are dominant and less polymorphic with a maximum number of two alleles at each locus (Jarne and Lagoda, 1996; Mueller and Wolfenbarger, 1999). Similarly, SSR markers revealed higher levels of genetic diversity than AFLP markers in the present study. However, the genetic diversity values obtained from both markers were highly correlated in this study, suggesting that both markers are neutral and reveal similar evolutionary processes acting on genetic diversity (Amos and Harwood, 1998). The high correlation of diversity estimates for the eight demes between two different types of genetic markers suggests a similar response of both marker types to the sampling strategy and the population history of the source populations. Since the genomic origin of both markers suggests the neutrality of most loci, we conclude that our results concerning the levels of diversity within demes appropriately reflect “neutral” diversity patterns, and, hence, the diversity at a large part of the genome. However, genomic regions under selection in certain demes or throughout the range of sampled populations might differ from the diversity patterns observed in this study.

We propose that an assessment of genetic diversity effects at the ecosystem level should be based on measurements characterizing intraspecific variation independent from specific plant traits. Accordingly, estimates of neutral genetic diversity are better suited to compare the magnitude of possible effects on different traits or interactions with accompanying organisms rather than diversity estimates within genes putatively involved in specific responses. The patterns of genetic diversity and differentiation reported here can later be compared to effects of the variation within and among the same demes at functional, phenotypic traits to search for possible correlations.

The genetic variation of the eight demes is different. Both SSR and AFLP markers show significant differences with regard to levels of genetic diversity. The *P. tremuloides* deme had higher genetic diversity than any other deme, including the population samples G1 and S, both at SSRs and AFLPs. This might be related to the different species status of the demes: genetic diversity in *P. tremuloides* (Wyman et al., 2003) might be higher than in *P. tremula* natural populations (Suvanto and Latva-Karjanmaa, 2005).

The seven *P. tremula* demes also had different levels of genetic diversity. The genetic diversities are strongly affected by the mode of sample collection (seeds from single seed parents, seeds sampled from few or numerous trees, or wildlings). As expected, the demes propagated from seeds of only one (G2, G8) or presumably few (CH, A) seed parents showed lower genetic diversity compared to demes collected as population samples (PL) or wildlings (S). But notably this was not found for G1, which was also supposed to originate from population sampling. Overall, considerable genetic diversity and in particular high allelic multiplicity (number of alleles per locus; **Table 3**) was observed in all demes, including those originating from a single or few seed parents. Accordingly, a high diversity of pollen contributions and a large effective number of pollen parents is assumed for these trees.

The heterogeneity of the origin of the demes planted in this trial greatly contributes to the observed complexity. The trial contains clonal structures (only in one deme; S), single-tree progenies (G2, G8), samples from presumably few (CH, A) and many (S, PL) seed parents of *P. tremula* and, as a reference, a population sample of *P. tremuloides* (USA). Since material was sampled in different locations within Europe from Switzerland to Sweden, an influence of large-scale geographic variation patterns on diversity levels of the sampled material cannot be excluded. However, triploid clones, as identified in a survey of natural populations of aspen (*P. tremuloides*) in North America (Mock et al., 2008), were not detected here. Because aspen can propagate by root suckers (Dickmann and Kuzovkina, 2008) the finding of some clones in the collection of wildlings from Sweden may not be unexpected. The genetic diversity of the Swedish deme was nevertheless only marginally affected by clonal structures, since diversity estimates were similar for the deme taking all trees, including clones, into account or after removing the clonal replicates prior to the calculation of genetic structures (**Table 3**). Furthermore, the genetic diversity observed in the Swedish deme is comparable to the diversity found in other natural populations of *P. tremula* in Sweden (Suvanto and Latva-Karjanmaa, 2005). Overall, the occurrence of clones reflects a natural situation for aspens and must be considered in diversity studies.

### GENETIC DIFFERENTIATION AMONG DEMES

Both SSR and AFLP markers showed significant differentiation among demes used in this study. It should be noted that the high variation of SSR loci within populations reduces estimates of genetic differentiation based on *F*_ST_ and related measures quantifying the proportion of the total diversity due to differences among populations (Hedrick, 2005). As expected, the highest differentiation was found between the North American *P. tremuloides* deme and the European *P. tremula* demes. Many private alleles in the *P. tremuloides* deme versus the *P. tremula* demes contribute to the difference between the two species. In addition, large morphological differences (in leaves and height) and phenological traits (leaf abscission) between the two species have also been found in this experiment (Kleemann, 2010; Müller et al., 2012).

Pronounced differentiation was also found among the *P. tremula* demes, which was even higher than that observed in natural populations of *P. tremula* (Hall et al., 2007). The reason for this finding was the heterogeneous origin of the planted demes. High differentiation between the studied demes follows from the complexities of sample collections and sources of demes. As expected, different types of sample collections influence the genetic differentiation in the present study.

### WITHIN-PLOT GENETIC DIVERSITY IN THE EXPERIMENT

For SSR markers, the genetic diversity for all plots was estimated as expected heterozygosity, which is the most commonly used parameter for co-dominant markers to estimate the level of genetic diversity in population genetics (Weir, 1996). For AFLP markers, the genetic diversity for all plots was estimated as Shannon index based on the molecular phenotypic diversity (band frequencies) since the estimation of allele frequencies is unreliable for several of the demes due to the violation of Hardy-Weinberg assumptions for progenies from a single or few seed parents.

The genetic diversities of plots comprising a given number of demes are highly variable. Thus, the genetic diversity of plots with a given number of demes is not homogeneous. This result is due to different diversity levels of the demes planted in the experiment and their complex patterns of (pairwise) differentiation. A particularly strong effect of increasing plot diversity was regularly observed if the USA deme (*P. tremuloides*) was included in mixtures of two or four demes. Both the high diversity of this deme and its strong differentiation from all other demes contribute to high diversity of plots containing this deme. Plots containing all eight demes consist of three plants from seven demes and four plants from one deme. Accordingly, the diversities of these plots are similar due to only minor differences of the contribution of the demes to these plots. Strong differences are particularly pronounced for two and four deme mixtures at AFLPs (**Figure 4**) due to the higher overall differentiation of AFLPs in comparison to SSRs. The diversity of these plots is considerably higher than the diversity of plots with the same number of demes but without *P. tremuloides*. The highest diversities were estimated for two-deme plots containing the Swedish and the USA (*P. tremuloides*) deme both at SSRs and AFLPs.

The average genetic diversity across plots is obviously increased with an increasing number of demes including or excluding *P. tremuloides*, thus, the number of demes is a proxy for the average genetic diversity of plots according to our results. However, the number of demes was not the key determinant of genetic diversity of single plot since, for example, the highest genetic diversity was not observed in the plot with the maximum number of demes, but in plots with 2-deme combinations including *P. tremuloides* and thus, was more strongly affected by inter-specific than by intra-specific genetic diversity. Thus, a prediction of genetic diversity within plots is highly unreliable if only based on the knowledge of the demes planted in the respective plot.

### GENETIC DIVERSITY IN RELATION TO INVERTEBRATE ABUNDANCE

Currently, bio-fuel plantations of *Populus* sp and *Salix* sp. are being expanded; they form habitats for numerous invertebrates implying problems to due leaf-feeding species but also providing beneficial ecosystem services (Landis and Werling, 2010). For examples, over 120 invertebrate species were recorded in the canopy of willows and poplars (Sage and Tucker, 1997, 1998). The insect-rich habitats of such plantations were used by birds as foraging sites (Sage et al., 2006). Furthermore, insects serve as pollinators in the surrounding landscape (Landis and Werling, 2010). Earlier studies have already shown strong relationships between plant species richness and insect species richness as well as their associated predators (Knops et al., 1999; Crutsinger et al., 2006). For example, a significant relationship between leaf feeding chrysomeloid species and leaf traits such as phenolic compounds in local *P. tremula* progenies was detected (Kleemann et al., 2011). Robinson et al. (2012) identified significant links between arthropod communities and poplar chemical traits in the Swedish SWAP collection. In contrast to those previous studies, here we focused on the assessment of “neutral” genetic diversity, since genomic regions involved in specific adaptive responses of poplar or related to species interactions or ecosystem functions such as productivity differ according to the traits involved and are rarely known (Neale and Ingvarsson, 2008; Dillen et al., 2009; Marron et al., 2010). Thus, these genomic regions are difficult to identify and usually relate to a single, specific interaction or function only, but not to others (Ingvarsson, 2005).

We expected that instead of testing a wide range of traits to identify relationships with important ecological functions such as the abundance of the associated invertebrate fauna, “neutral” genetic diversity as an integrative index for evolutionary adaptation might be suitable to depict significant relationships. However, the correlation uncovered here for neutral genetic diversity and invertebrate abundance was only significant, if the plots with *P. tremuloides* were included in the analysis. Because the diversity range covered by plots containing different *P. tremula* demes was almost twice as high as that covered by the plots with *P. tremuloides*, our data suggest that interspecific differences in plots may be more important for this relationship than intraspecific differences. Nevertheless, it is interesting that we found a positive relationship with total invertebrate abundance and a negative with caterpillars which are feeding on the leaves. A drawback of our data is that we did not distinguish in the group of beetles herbivores and other guilds. However, most of the beetles that occur on young aspen are poplar leaf beetles (*Chrysomela populi*, Kleemann et al., 2011, Volmer, personal observation). These beetles cause massive feeding damage on young leaves and thus, can lead to significant economic loss in poplar plantations (Urban, 2006; Fernandez and Hilker, 2007). Here, a moderate significant increase in beetle abundance was observed in 4-deme compared 2-deme or 1-deme plots supporting that the genotypic composition of the aspen is relevant for beetles (Kleemann et al., 2011). Usually, genetic uniformity of typical biomass plantations, consisting of only one distinct, high-yielding clone, renders them relatively sensitive to infestation by herbivores or other pests. The present data suggest that introducing increased genetic diversity into these plantations is *per se* insufficient to manage canopy fauna. However, it was interesting that the abundance of beetles was lower on the plots with the European aspen than on those with the USA deme, which may indicate that the former were better protected from potentially leaf-feeding insects. Because insects serve as food for higher trophic levels and can affect the surrounding landscape as pollinators, they are important regulators of ecosystem services. It is obvious that further and more detailed studies are needed to investigate the relationship between genetic diversity and ecological services. The current study highlights the need to distinguish between the relative importance of traits (i.e., distinct gene loci) and neutral genetic diversity as basis to drive ecosystem functions.

## CONCLUSION

A better understanding of the role of intraspecific diversity for ecosystem processes and services requires the establishment of experimental populations under conditions closely resembling natural ecosystems. While it is straightforward to manipulate clonal diversity in woody species, only few forest trees regularly propagate as clones. Therefore, the manipulation of intraspecific diversity in experimental tree populations using sexually produced progenies is needed to better understand links between evolutionary and ecological processes. Our field study is one of the first experiments in this regard. Its genetic diversity was assessed ex post. Thus, the genetic diversities of the plots were unknown at the time of trial establishment and could not be modified later. A thorough assessment of genetic diversity prior to the establishment of a trial will allow an optimization of the trial design with regard to the different intraspecific diversities represented in the plots and their spatial arrangement. Our results indicate that genetic diversity within different aspen demes may exhibit significant differences. Furthermore, mixing different demes increases the genetic diversity, however, the magnitude of this effect is small compared with the diversity present in distinct combinations. Highest diversity was present in mixtures of *P. tremula* with *P. tremuloides*. To address the difference between inter- and intraspecific genetic diversity future studies should include the same number of demes for different species.

In this study the intraspecific diversity patterns offer unique opportunities to better understand the role of genetic variation on ecosystem processes and services, and are an interesting case to study the complexity of diversity triggered by sexual reproduction in experimental plant populations. Detailed morphological, physiological, and molecular studies on poplars and associated organisms are needed to understand impacts of intraspecific diversity on ecosystem processes and functions. However, the interpretation of the variability at these traits must rest on a solid analysis of their genetic basis.

## AUTHOR CONTRIBUTIONS

Chunxia Zhang conceived the study, participated in its design, carried out the molecular genetic studies, performed the statistical analysis and drafted the manuscript. Barbara Vornam and Kathleen Prinz participated the data analysis and helped to draft the manuscript. Katharina Volmer carried out invertebrate collection in the field, participated the statistical analysis and helped to draft the manuscript. Frauke Kleemann conceived the experiment, carried out the seeds or seedlings collection and cultivation and planted seedlings in the experimental field and helped to draft the manuscript. Lars Köhler participated in the design of the POPDIV and coordination and helped to draft the manuscript. Andrea Polle conceived and designed the POPDIV, analyzed data and drafted the manuscript. Reiner Finkeldey conceived the study, participated in its design, the data analysis and drafted the manuscript. All authors read and approved the final manuscript.

## Conflict of Interest Statement

The authors declare that the research was conducted in the absence of any commercial or financial relationships that could be construed as a potential conflict of interest.

## Footnotes

1 http://www.r-project.org/

2 http://folk.uio.no/ohammer/past/
